# Should Attractive Males Sneak: The Trade-Off between Current and Future Offspring

**DOI:** 10.1371/journal.pone.0057992

**Published:** 2013-03-13

**Authors:** Ulrika Candolin, Leon Vlieger

**Affiliations:** Department of Biosciences, University of Helsinki, Helsinki, Finland; University of Melbourne, Australia

## Abstract

Alternative reproductive tactics are predicted to be adopted by less competitive males when competition for fertilization is intense. Yet, in some species, competitively superior males use an alternative tactic alongside the conventional tactic. This can jeopardize their success through the conventional tactic, but surprisingly little attention has been paid to this cost. We investigated 1) the degree to which competitive males sneak fertilize eggs in the polygamous threespine stickleback, *Gasterosteus aculeatus*, and 2) if males balance the cost of sneaking against its benefit. We found competitive males that succeeded in establishing a territory and in attracting spawning females to perform most sneak fertilizations. However, when we reduced the benefit of sneak attempts, by reducing visibility and the success rate of sneak attempts, males sneaked less. When we increased the cost of sneak attempts, by increasing the perceived value of current offspring (by mating males to preferred females rather than unpreferred females or no females), the interest of males in sneak opportunities decreased. Intriguingly, larger males, who presumably had a higher probability of future reproduction, were more willing to risk their current offspring for sneak opportunities. These findings suggest that competitive males that are attractive to females carefully balance costs against benefits in their sneaking decisions. More broadly, our results imply that changes in the environment can influence the cost-benefit ratio of sneaking and alter the distribution of fertilizations in a population. We end with discussing the implications that alterations in sneaking behavior could have for the operation of sexual selection in changing environments.

## Introduction

Males with a low probability of gaining fertilizations through a conventional tactic, such as courtship or fighting, are predicted to employ an alternative tactic, such as cuckoldry or sneaking [1]–[5]. The choice of a tactic is based on social and environmental cues, with males of high status or condition adopting the conventional tactic because they receive higher fitness benefits from this. The tactics thus form part of a conditional strategy [1], [3] with equal or unequal average fitness benefits [3], [6], [7]. An example is male natterjack toads, *Bufo calamita*, that adopt a non-calling satellite tactic when close to males emitting high-quality advertisement calls [8]. The conditional strategy differs from an alternative strategy where alternative phenotypes are genetically determined and have equal average fitnesses, maintained by frequency-dependent selection [1]. For instance, male side-blotched lizards, *Uta stansburiana*, have three genetically determined alternative strategies, which are maintained by a rock-paper-scissor dynamic [9].

In some species, competitively superior males can adopt an alternative tactic opportunistically, alongside the conventional tactic [5]. For example, nesting red-winged blackbird males, *Agelaius phoeniceus*, often sire offspring in the nests of neighboring males [10], [11]. The evolution of the propensity to use both tactics simultaneously is favored whenever the fitness gain of using both tactics is higher than the fitness loss. Yet, investigations have concentrated on the consequences of male alternative tactics for females [12]–[14], while little attention has been directed to the trade-off that males face between cost and benefits of employing an alternative tactic and how the optimal allocation depends on the context.

Alternative tactics are less expensive than conventional tactics [15], but their cost increases if they are employed alongside the conventional tactic, as they can reduce the success of the conventional tactic. For instance, pair forming male birds that engage in extra-pair copulations often suffer from cuckoldry, female divorce and sperm depletion that reduce their reproductive success with the social partner [16]. Thus, males have to carefully balance the benefit of employing an alternative tactic against the cost, i.e. they have to balance the probability of reproductive success through the alternative tactic (the fitness gain) against the probability of losing current and/or future reproductive success (the fitness loss). The optimal allocation then depends on environmental conditions and intrinsic properties of the male.

Here, we investigated 1) the degree to which competitive, resource-holding males of a polygamous fish use an alternative tactic alongside the conventional, resource-holding tactic, and 2) if this is adjusted to context-dependent costs and benefits of employing the alternative tactic. As model organism, we used the threespine stickleback, *Gasterosteus aculeatus,* which has two conditional tactics: courtship and sneaking. Courtship is performed by males that have established a territory and built a nest, while sneaking is performed by both nesting and non-nesting males, by parasitizing on the courtship effort of nesting males and attempting to sneak fertilize any eggs spawned into the nest [17]–[19]. Nesting males care alone for the developing embryos in the nest.

The courtship tactic is costlier than the sneaking tactic in terms of energy expenditure [20], but also more rewarding as females only spawn in nests of courting males [17]. The cost of sneaking is expected to differ between nesting and non-nesting males, with the cost being higher for nesting males. Non-nesting males only risk future reproductive success, including the probability of becoming a nesting male, because of time and energy spent on sneaking, while nesting males risk both current and future reproductive success when leaving their nest unattended. A nesting males has to regularly fan oxygen rich water into the nest [17], [19], [21]–[23] and defend his territory and any developing embryos against intruders and predators [17], [24], [25]. Nesting and non-nesting males also differ in how the cost of sneaking varies over the season. Nesting males reproduce repeatedly during a single breeding season [26] and the cost of leaving the nest increases over the season when the probability of replacing current offspring declines, while the cost of sneaking decreases over the season for non-nesting males – who do not care for offspring – when future reproductive opportunities decline. Seasonal changes in reproductive opportunities are known to influence reproductive decisions, across taxa [27], but little attention has been paid to the possibility of males adjusting their sneak decisions to future reproductive opportunities.

We determined the sneaking rate of nesting and non-nesting threespine stickleback males by allowing groups of males to build nests and spawn with females in experimental pools. Fertilization success was determined through molecular parentage analysis. To investigate if males balance the cost of sneaking against the benefit, we manipulated the benefit of sneak attempts by altering the density of artificial vegetation. This reduces visibility and hampers the detection of sneak opportunities and decreases sneaking success [28]. To examine if the cost of sneaking, in terms of the loss of current reproductive success, influences sneak attempts, we performed a separate experiment where we manipulated current reproductive success, by allowing males to spawn with preferred females, unpreferred females or no females, and noted their willingness to leave the nest unattended to inspect a sneak opportunity.

## Methods

### Ethics Statement

The experimental procedures comply with the laws of the country in which they were performed. They were approved by the Animal Care Committee of the University of Helsinki (86-06) and by the National Animal Experiment Board in Finland (STH421A).

### Fish Collection and Maintenance

We collected sticklebacks with minnow traps in early May before the start of the breeding season from a bay close to Tvärminne Zoological Station in the Baltic Sea (60°N, 23°E). The fish were transported in aerated tanks to the station and housed in flow-through holding tanks, at a density of 0.25 fish/liter, sexes separated, under natural light and temperature conditions in an outdoor facility. The fish were fed daily on frozen chironomid larvae.

### Pool Experiment: Sneaking and Habitat Structure

To determine the sneaking rate of nesting and non-nesting males and its dependency on habitat structure and visibility, we allowed groups of eight males to breed in wading pools (N = 40), 1.8 m in diameter, with a low or a high density of artificial vegetation. The size range of the fish (44 to 63 mm standard length, SL) was the same across pools (mean body size ± SE: sparse vegetation: 51.1±0.4 mm, dense vegetation: 51.2±0.4 mm). All males were sexually mature, as revealed by their blue eyes and hints of red nuptial coloration. Pools with a low density of vegetation (N = 20) had four bunches of 15 cm long, thin, green polypropylene strings [29] evenly distributed over the bottom, while pools with a high density of vegetation (N = 20) had 17 bunches of polypropylene strings distributed over the bottom. The males were individually marked by clipping the tip of the three dorsal spines in a unique combination before being placed in the pools. All males experienced the same handling procedure and they resumed normal swimming behavior within a few minutes. The experiment was run during the height of the breeding season, from end of May to mid-June. The density of males and vegetation corresponds to natural conditions in the field [30].

The males were observed twice a day for 10 min, in the morning and in the afternoon, during 2 days. This allowed us to determine which males established territories and built nests. Territorial males are easily recognized by their aggressive behavior against other males. To confirm the identity of the territorial males, they were dip-netted and inspected after the last observation. This took only a few seconds and all males resumed normal territorial behavior within less than 5 min. On the third day, four gravid females were sequentially added to each pool, at intervals of two hours. This reflects the natural mate encounter rate in the field [30]. The behavior of each female was observed until spawning occurred or for a maximum of one hour.

Two days later, all nesting males were dip-netted from their territories and their identity checked, after which their nests and the remaining males and females were collected. Egg clutches originating from different females could be visually separated within the nests, as the clutches differed slightly in color and were partly separated. The divisions were confirmed by parentage analyses. The total weight of each egg clutch was measured by weighing the eggs to the nearest 0.01 g. The weight of developed eggs was measured after removing undeveloped eggs. Developed eggs (embryos) and a tail fin clip from each fish were preserved in 96% ethanol for parentage analysis. The fish were humanely killed through decapitation. At least 20 embryos were analyzed for each clutch. Females in the current population usually lay 50–200 eggs per spawning (unpublished material). The eggs were collected from different parts of the clutch to increase the probability of detecting sneak fertilizations. To validate the method, we analyses all eggs in five egg clutches, containing between 119 and 192 eggs (mean = 149, SE = 14), for which sneaking had been observed. Our method detected all sneak events but slightly overestimated the proportion of eggs sneak fertilized for larger egg clutches (linear regression of estimated proportion on actual proportion of sneak fertilized eggs, r^2^ = 0.99, Y = 0.003 (SE = 0.010) +1.14 (SE = 0.05) X. Because the size of the egg clutches sneak fertilized did not differ between nesters and non-nesters (mixed model with pool as random factor, F_1,9_ = 0.01, P = 0.93), or between treatments (F_1,19_ = 2.07, P = 0.17), the slight overestimation did not bias our results. The proportion of the eggs that each male fertilized was transformed to amount of eggs fertilized, by multiplying the proportion with the total amount of developed eggs in the clutch. In the analyses, we included only pools for which the parentage of the eggs could be determined, resulting in 17 pools with sparse vegetation and 19 pools with dense vegetation, with a total of 288 males.

Females spawn all ovulated eggs at one spawning, into one nest, and cannot divide the eggs among nests [17]. This allowed us to separate between sneaking and egg stealing. When the eggs of one female were fertilized by several males, sneaking had occurred. When the eggs of one female were found in more than one nest, or all her eggs were in the nest of a male with no paternity, then egg stealing had occurred. This assumes that no nesting males were sterile. Sterility is uncommon among territorial stickleback males with nuptial coloration (Candolin, personal observation). When two or more nesting males had fertilized the same clutch of eggs, fertilizations were assumed to have taken place in the nest of the male with the highest paternity. This was based on the observation that the nest owner always swims through the nest and fertilizes the eggs before the sneaker [28]. It was confirmed by direct observation of 14 sneak spawnings in which the nest owner fertilized the majority of the eggs. Egg cannibalism could have influenced our measure of fertilization success. However, the total amount of eggs collected from the pools did not differ between treatments (sparse vegetation, mean ± SE: 1.27±0.07 g, dense vegetation: 1.20±0.10 g, t_34_ = 0.30, P = 0.59), which suggests that cannibalism did not differ between treatments. All females had spawned their eggs and the females did not differ in the loss of body weight during spawning (sparse vegetation: 0.36±0.01 g, dense vegetation: 0.35±0.02 g, mixed model with pool as random factor, F_1,34_ = 0.37, P = 0.54). Based on the parentage analysis, we determined the amount of eggs each male gained through courtship and the amount of eggs each male fertilized through courtship and through sneaking.

When analyzing the data, we used linear and non-linear mixed-models (REML) with pool as a random factor to consider dependencies within pools. When the response variable was binary, we used GLMM and the “lmer” function together with a logit link from the lme4 package of the software R 2.11.1 (R Development Core Team 2010). For pool averages, we used linear models. The normality of the residuals from the models were checked visually (qq and pp-plots) and statistically (Shapiro–Wilk test). The percentage of the amount of eggs sneak fertilized and the percentage that was stolen were log (x +1) transformed to reach normality. Male length and weight were strongly correlated and only male weight was used as a measure of male size.

### Molecular Parentage Analyses

Adults and eggs were genotyped using six microsatellite loci (*STN21, STN57, STN163, STN110, STN174, 7033PBBE*) (information on the loci, the DNA extraction procedures and the polymerase chain reaction conditions are given in File S1). Parentage was assigned using the program CERVUS 3.0 [31], simulating genotypes for 100,000 offspring per mating and then running parent pair analyses with known sexes. Only samples for which parentage was assigned with 95% confidence were included. Parent-embryo mismatches occurred for one replicate and were consistent with the occurrence of a null allele at STN57. Parentage analysis for this replicate was repeated without the locus. All analyses were repeated to confirm the reliability of the results.

### Aquarium Experiment: Influence of Offspring Value

**Figure 1:**
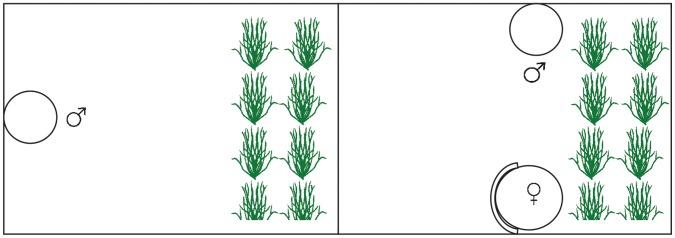
The experimental aquarium with the focal male to the left.

To investigate if males trade costs against benefits in their sneaking decisions, we determined the influence of the value of current reproductive success on a male’s willingness to leave his nest unattended and inspect a courting male. Sneaking males usually inspect a courting pair for several minutes, and females often leave without spawning. To manipulate current reproductive success, we allowed individual males, whose length (±1 mm) and weight (±0.01 g) had been measured, to build a nest on a nesting dish [32] in individual 10-l aquaria. When a male had completed nest building, we moved him, with the nesting dish, to one of the short ends of an experimental aquarium (60×40 cm, Fig. 1). At the other end of the aquarium was a vegetated area (20×40 cm). After one day of acclimatization, we placed two size-matched, gravid females, enclosed in perforated Plexiglas cylinders (diameter 12 cm), 30 cm from the male’s nest, and 16 cm from each other. We allowed the male to court the two females for 15 min. If the male showed a clear preference for one of the females, by courting her at least 75% of the courting time, we submitted him to one of the following treatments: 1) both females removed, 2) preferred female removed and the non-preferred female released, 3) non-preferred female removed and the preferred female released. We allowed the released female to spawn in the nest of the male, after which we removed her.

Two hours after the removal of the female, we removed an opaque divider at the vegetated end of the aquarium and allowed the male to view a similar aquarium, containing a nesting male of the same size as himself. The nest of the neighboring male was 30 cm from the focal male’s aquarium (Fig. 1). The focal male could observe the neighboring male while hiding in the vegetated part of his aquarium, while the ability of the neighboring male to observe the focal male was hampered as the focal male was concealed by the vegetation. After one hour, we placed a gravid female, enclosed in a Plexiglas cylinder (12 cm in diameter), 20 cm from the neighboring male’s nest and 30 cm from the focal male’s aquarium. The cylinder was opaque at the side facing the focal male, mimicking a stone concealing the female, to prevent interactions between the female and the focal male. The focal male could inspect the neighboring male courting the female, and, thus, observe an opportunity for sneaking. Whether the male would have sneaked if allowed to is unknown. During 15 min we recorded the number and duration of visits by the focal male to a 10 cm zone next to the neighboring aquarium. Finally, we measured the amount of eggs in the focal male’s nest by weighing the clutch of eggs. We performed 23 replicates of each treatment. After the experiment, the fish were released at the site of capture.

## Results

### Pool Experiment: Sneaking and Habitat Structure

**Figure 2:**
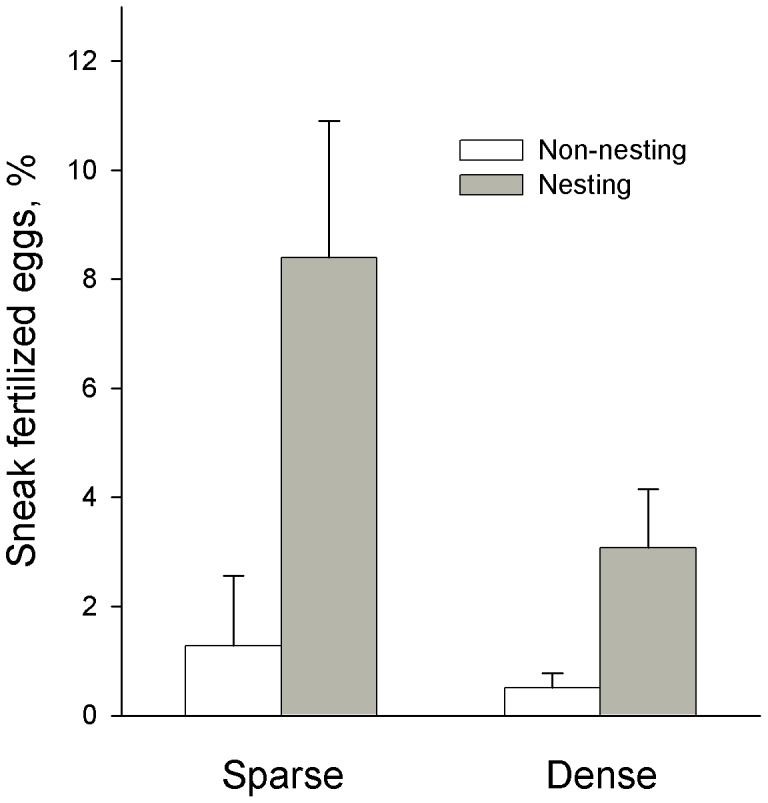
Mean (+ SE) proportion of eggs that were sneak fertilized by non-nesting and nesting males, in pools with sparse and dense vegetation. Untransformed values are presented.

More males nested in pools with dense vegetation (mean ± SE: 4.2±0.3) than in pools with sparse vegetation (3.1±0.2, F_1,34_ = 8.62, P = 0.006). In both treatments, the nests were evenly distributed within the pools, apparently maximizing the distance between them. The males that nested tended to be heavier than males that did not nest, independent of vegetation (mixed model: F_1,273_ = 2.82, P = 0.094). The number of males that received eggs through courtship did not differ between vegetation treatments (sparse: 1.9±0.2, dense: 2.3±0.2, F_1,34_ = 2.06, P = 0.16), but a smaller proportion of the spawned eggs were sneak fertilized in dense vegetation (F_1,34_ = 4.49, P = 0.041, Fig. 2). Nesting males sneak fertilized a larger proportion of the eggs in a pool than non-nesting males and the difference was more pronounced in sparse vegetation (mixed model: nesting status: F_1,284_ = 15.83, P<0.001, nesting status*vegetation: F_1,284_ = 5.62, P = 0.018, Fig. 2). The proportion of the eggs in a pool that were stolen did not differ between vegetation treatments (untransformed values: sparse: 16.5±6.7%, dense: 5.9±2.5%, log+1 transformed values: F_1,34_ = 2.23, P = 0.14).

**Figure 3:**
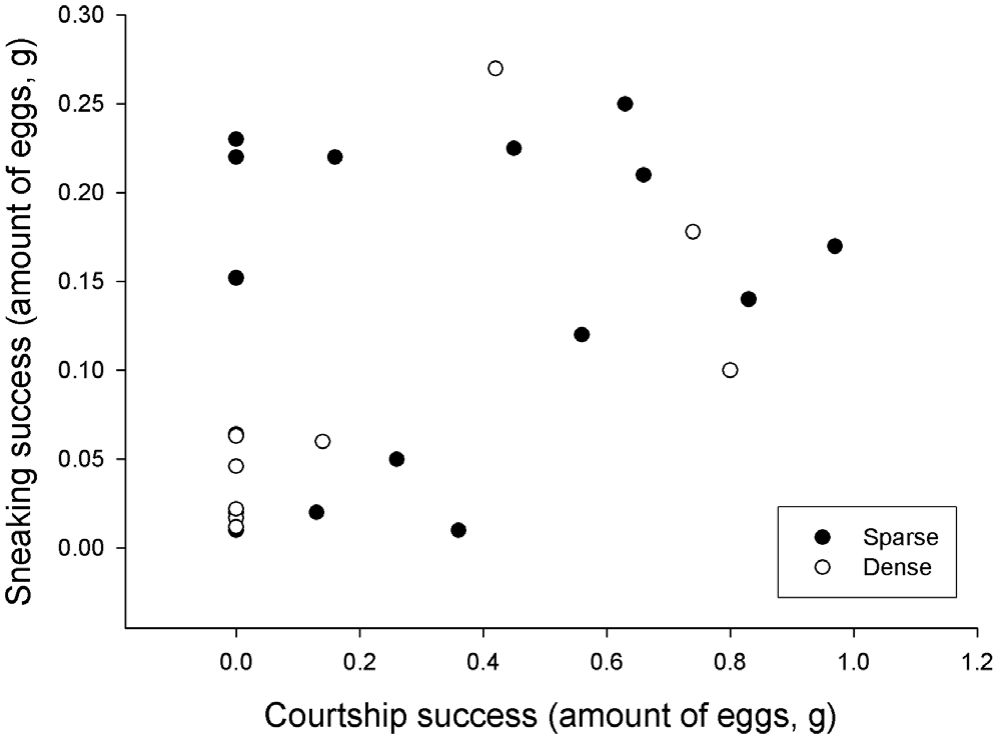
Correlation between amount of eggs sneak fertilized and courtship success in sparse and dense vegetation. Dependencies within pools are not shown.

The males that sneaked had a higher total fertilization success than males that did not sneak and the pattern was more pronounced in sparse vegetation (mixed model: sneaking: F_1,284_ = 14.57, P<0.001, sneaking*vegetation: F_1,284_ = 4.69, P = 0.031). The amount of eggs a male fertilized through sneaking correlated with the amount of eggs the same male fertilized through courtship, independent of vegetation (mixed model including only males that sneak fertilized eggs: F_1,25_ = 7.51, P = 0.011, Fig. 3).

The males that sneaked did not differ in body size from the males that did not sneak, independent of vegetation (mixed model: F_1,281_ = 0.98, P = 0.32). Among nesting males, the males that sneaked did not differ in size from the males that did not sneak, independent of vegetation (mixed model: F_1,124_ = 0.23, P = 0.63). Nesting males that sneaked tended to be the victims of sneaking less often than nesting males that did not sneak, independent of vegetation (GLMM with binomial response variable, N = 132 nesting males, z = 1.87, P = 0.061). The victims of sneaking did not differ in size from nesting males that were not the victims, independent of vegetation (F_1,126_ = 0.43, P = 0.51).

### Aquarium Experiment: Offspring Value

**Figure 4:**
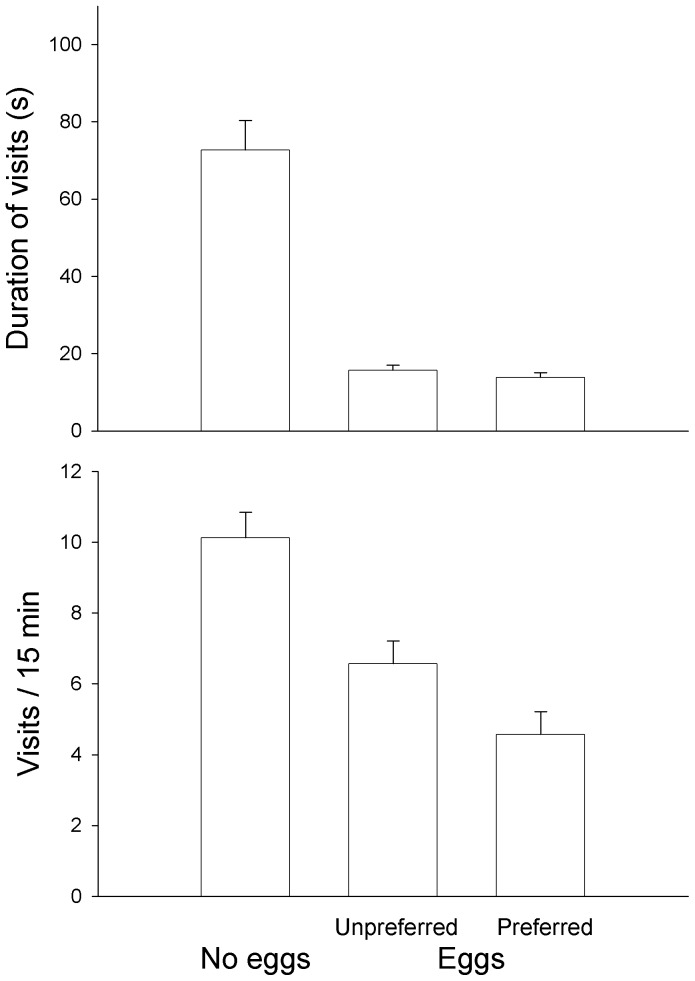
Mean (+ SE) number and duration of inspections by males without eggs and by males with eggs of unpreferred and of preferred females.

Males with eggs in their nest inspected the courting male less often (F_1,66_ = 29.74, P<0.001) and for shorter times than males without eggs (F_1,66_ = 106.43, P<0.001, Fig. 4). One male did not inspect the courting male at all. Males with eggs of a preferred female inspected the courting male less often than males with eggs of an unpreferred female (F_1,44_ = 4.90, P = 0.032), but the males did not differ in the mean duration of the inspections (F_1,43_ = 1.05, P = 0.31, Fig. 4). The amount of eggs in the nest did not differ between males receiving eggs from a preferred or an unpreferred female (F_1,44_ = 1.25, P = 0.27). The duration of the inspections did not correlate with the amount of eggs received, independent of which of the two females had spawned the eggs (F_1,43_ = 0.09, P = 0.76).

Among males with eggs, male size correlated positively with the number of inspections, independent of which female had spawned the eggs (F_1,44_ = 6.11, P = 0.017), but male size did not correlate with the duration of the inspections (F_1,43_ = 1.93, P = 0.17). No relationships between male size and number and duration of inspections were detected for males without eggs (both P>0.1).

## Discussion

Our results show that nesting threespine stickleback males use an alternative sneaking tactic alongside the conventional courtship tactic, but that the employment of the tactic is adjusted to both current and expected future reproductive success. The pool experiment showed that in a group of both nesting and non-nesting males, nesting males performed most sneak fertilizations, with attractive males with a high courtship success sneaking the most, but decreasing sneaking in dense vegetation where the success rate of sneak attempts was low. The aquarium experiment revealed that males were more willing to risk their current reproductive success for a sneak opportunity when the perceived value of current offspring was low, but that risk taking increased with male body size, which presumably correlated with future reproductive opportunities [33]. These results suggest that males adjust sneaking to its context-dependent costs and benefits.

Why did nesting males perform most of the sneak fertilizations? Little is known about the sneaking behavior of resource holding males across taxa, but three non-mutual possibilities exist: 1) territoriality, or traits associated with territoriality, promoted sneaking, 2) females preferred to spawn in the presence of sneaking nesters, or 3) non-nesting males invested less in sneaking because they were saving resources for territoriality and future reproductive opportunities. Regarding the first possibility, it is conceivable that traits related to territoriality, such as boldness [34], increased the inclination of males to search for sneak opportunities, or increased their sneaking success, as sneaking could be part of a behavioral syndrome with consistent correlations between traits [35], [36]. In a few other species, the employment of an alternative tactic has been found to correlate with territorial behavior, such as in red foxes, *Vulpes vulpes*, where males with larger territories sire more extra-pair offspring [37].

The second possibility, that females preferred to spawn in the presence of sneaking nesters, could hold if females were able to detect the sneaker and perceive his nesting status. This appears unlikely considering the inconspicuous habitus of sneakers, but cannot be excluded. Alternatively, females could have preferred to spawn with males surrounded by attractive, nesting males. Whether females should favor or avoid sneakers depends on the genetic quality of the sneakers, their fertilization success, and the influence of sneaking on paternal care [38]. The present results suggest that females benefit from sneaking, as sneakers often were attractive, preferred males. These could be of high phenotypic and/or genetic quality and, hence, provide superior direct benefits - such as fertilization success – and/or superior indirect, genetic benefits. Moreover, females could reduce the risk of cannibalism by neighboring males by allowing these males to gain paternities through sneaking [39]. On the other hand, sneaking could reduce male parental investment and, thus, the fitness benefit of sneak fertilizations [40]. Yet, in a few fishes, females prefer to spawn in the presence of sneakers [41]–[44], which suggests that the benefit of accepting sneakers can be higher than the cost. The generality of the pattern remains, however, to be determined.

The third possibility, that non-nesting males refrained from sneaking in favor of future nesting opportunities is also plausible, as the intensity of competition for nesting sites varies spatially and temporally under natural conditions [33]. The competitive ability and attractiveness of males could change over the season, particularly as they show indeterminate growth [45]. In general, males should employ a resource-holding tactic whenever the fitness gain of this is higher than the gain of the alternative tactic. For instance, subdominant males of the speckled wood butterfly, *Pararge aegeria*, abandon a patrolling tactic and adopt a territorial ‘perching’ tactic if given the opportunity [46].

Sneaking success was correlated with courtship success, which indicates that nesting males that sneaked were attractive males that further increased their fertilization success by parasitizing on the effort of neighboring males. Parallels can be drawn to birds where attractive males have a higher extra-pair fertilization success than less attractive birds [12]. However, female birds choose or accept their extra-pair mates while fertilization in sticklebacks takes place after the female has left the nest, without her consent [45]. It is possible that attractive stickleback males had a higher sneaking success than less attractive males because they performed more sneak attempts, due to higher benefits or lower costs of sneaking, or because they had a higher success rate per attempt, due to more sperm or more competitive sperm [47]. Attractive males could pay a lower cost of sneaking because of a higher probability of replacing lost offspring, or because they are dominant males able to replace a lost territory or chase away an intruder [48].

The aquarium experiment shows that the willingness of nesting males to leave their nest and spend time inspecting a sneak opportunity depends on the presence of eggs in the nest and their perceived value. Males with eggs left their nest less often and for shorter duration than males without eggs. This is probably because of the cost of sneaking in terms of the risk of losing current reproductive success. Interestingly, males mated to an unpreferred female left their nest more often than males mated to a preferred female. Thus, risk-taking appeared to be adjusted to small scale differences in the cost of sneaking. Alternatively, a difference in the need of parental care could have influenced the frequency of inspections. However, this seems unlikely as males with eggs of preferred and unpreferred females did not differ in the amount of eggs in their nests or in the duration of the inspections. The low interest of parental males in sneak opportunities appears at first sight to conflict with the pattern detected in the pool experiment, where males with a high courtship success sneaked the most. However, courtship success in the pools depended on intrinsic properties of the males, while courtship success in the aquarium experiment was randomly assigned by us. Thus, the presence of eggs in the nest in the aquarium experiment did not correlate with male attractiveness or competitive ability, and, hence, not with the males’ intrinsic inclination for sneaking.

In the aquarium experiment, risk taking did depend on the probability of future reproduction, as larger males with a higher likelihood of replacing lost offspring inspected the courting male more often than smaller males. Larger males are preferred by females [49] and more successful at establishing territories [33] and should experience a lower cost of sneaking. An alternative explanation is that larger males were more inclined to chase away the neighbor, or to occupy his territory, and therefore visited the border more often. However, this appears unlikely as the males and the territories were size matched and the border of vegetation prevented one male from merging the two territories [30]. In the pool experiment, no correlation between male size and sneak fertilization was detected, which suggests that other factors determined ultimate sneaking success. For instance, larger males could have a lower success rate per sneak attempt.

Sneak fertilizations were less common in dense vegetation, for both nesting and non-nesting males. This could be a consequence of a lower success rate per sneak attempt [28] or of fewer sneak attempts, as reduced visibility hampers the detection of a courting male [23], [50], [51] and restricts the movements of territorial males [30]. Reduced sneaking could, in turn, relax sexual selection, as sneaking was mostly performed by attractive males with a high courtship success. This could contribute to the general relaxation of sexual selection in eutrophied environments [52]. The consequences that the relaxed sexual selection could have for the viability of populations, through effects on direct benefits of mate choice and the good genes process, would deserver further investigations [53]–[55].

To summarize, we found nesting males with a high courtship success to perform most sneak fertilizations. Sneaking, and the interest of males in sneak opportunities, was adjusted to current reproductive success and the probability of future reproduction. Thus, resource-holding males appeared to carefully balance costs against benefits in their sneaking decisions. We further found increased vegetation cover to reduce sneaking. This could relax sexual selection on traits, which highlights the potential importance of alternative reproductive behaviors in mediating impacts of environmental change on evolutionary processes.

## Supporting Information

File S1 **Molecular parentage analyses: DNA extraction, the polymerase chain reaction conditions and the microsatellite loci.**
(DOC)
