# Supplementary material for: Should Attractive Males Sneak: The Trade-Off between Current and Future Offspring
Source: PLoS One. 2013 Mar 13;8(3):e57992. doi: 10.1371/journal.pone.0057992 (PMC3596318; doi:10.1371/journal.pone.0057992)
Supplement: File S1 — Molecular parentage analyses: DNA extraction, the polymerase chain reaction conditions and the microsatellite loci. (DOC) [file pone.0057992.s001.doc]

**File S1.**

**Molecular parentage analyses: DNA extraction, the polymerase chain reaction conditions and the microsatellite loci**

DNA was extracted from eggs and tail fins by proteinase K digestion in 5% Chelex 100 resin (Bio-Rad Laboratories, Hercules, CA). For fins, this was followed by a purification step by means of vacuum filtration on a 96-well 0.45 μm GHP AcroPrep filter plate (Pall Life Sciences, Ann Arbor, MI).

To visualize polymerase chain reaction (PCR) products, the forward primer was labelled with a fluorescent dye (FAM, HEX or TET) and the 5’-end of the reverse primer modified with a GTTT-tail to enhance 3’-adenylation . DNA was amplified in a 10 μl reaction volume containing 1.5x PCR reaction buffer (160 mM (NH_4_)_2_SO_4_, 670 mM Tris-HCl (pH 8.8), 0.1 % Tween-20 (Bioline, London, UK), 1.5 mM MgCl_2_ (Bioline, London, UK), 0.2 mM dNTP (Finnzymes Oy, Espoo, Finland), 0.25 U Bio*Taq* DNA polymerase (Bioline, London, UK), 2-5 pmol of each primer, and approximately 5 ng of template DNA. Thermal cycling was done using the following protocol: 95°C for 3 min, 35× (95°C for 30 sec, 53°C for 30 sec, 72°C for 30 sec), 72°C for 5 min. PCR products were diluted 1:100 with MQ-water and 3 μl mixed with 12 μl ET-ROX 400 size standard (GE Healthcare, Buckinghamshire, UK) prior to injection in a MegaBACE 1000 automated sequencer (GE Healthcare, Buckinghamshire, UK). Genotypes were scored with the program Fragment Profiler 1.2 (GE Healthcare, Buckinghamshire, UK) and by visual inspection and manual correction of the allele peak data.

**Table S1. The six microsatellite loci used in the paternity analyses.**

| Locus name | GenBank Accession no. | MP | Size range (bp) | *H_e_* | Alleles |
| --- | --- | --- | --- | --- | --- |
| STN21 | G72136 | a | 132-226 | 0.96 | 42 |
| STN57 | G72155 | a | 101-179 | 0.96 | 35 |
| STN163 | G72304 | a | 133-201 | 0.90 | 34 |
| STN110 | G72182 | b | 158-194 | 0.88 | 19 |
| STN174 | G72310 | b | 91-147 | 0.87 | 24 |
| 7033PBBE | AJ010360 | b | 193-261 | 0.90 | 30 |

MP, multiplex PCR; *H_e_*, expected heterozygosity

STN loci were described in Peichel et al. .

PBBE loci were described in Largiadèr et al. .

**Literature cited**

Brownstein MJ, Carpten JD, Smith JR. 1996. Modulation of non-templated nucleotide addition by tag DNA polymerase: Primer modifications that facilitate genotyping. Biotechniques. 20:1004-1010.

Elphinstone MS, Hinten GN, Anderson MJ, Nock CJ. 2003. An inexpensive and high-throughput procedure to extract and purify total genomic DNA for population studies. Mol Ecol Notes. 3:317-320.

Largiader CR, Fries V, Kobler B, Bakker TCM. 1999. Isolation and characterization of microsatellite loci from the three-spined stickleback (*Gasterosteus aculeatus* L.). Mol Ecol. 8:342-344.

Peichel CL, Nereng KS, Ohgi KA, Cole BLE, Colosimo PF, Buerkle CA, Schluter D et al. 2001. The genetic architecture of divergence between threespine stickleback species. Nature. 414:901-905.
